# Determining the Effect of External Stressors and Cognitive Distraction on Microsurgical Skills and Performance

**DOI:** 10.3389/fsurg.2019.00077

**Published:** 2020-01-22

**Authors:** Shane Carr, Bronwyn Reid McDermott, Niall McInerney, Alan Hussey, D. Byrne, Shirley Potter

**Affiliations:** ^1^Department of Plastic and Reconstructive Surgery, Galway University Hospital, Galway, Ireland; ^2^Irish Centre for Applied Patient Safety and Simulation, Galway University Hospital, Galway, Ireland

**Keywords:** microsurgery, simulation, cognitive distraction, external stress, microsurgical skills

## Abstract

**Introduction:** Microsurgery is an essential element of Plastic Surgery practice. There is a paucity of studies assessing the impact of stress and cognitive distraction on technical microsurgical performance. The ability to complete cognitive and technical skills in parallel has not been assessed in a microsurgical setting.

**Aim:** To test the hypothesis that cognitive distraction and external stressors negatively affect microsurgical performance in a high fidelity simulation setting.

**Materials/Methods:** Fourteen surgeons across all levels of training undertook 2 microsurgical skills sessions, 1 month apart. Session one established baseline microsurgical skill. In session two, skills were assessed with the introduction of realistic operative room cognitive distractions (ORDIs). Outcome measures were efficiency and accuracy, measured by Time to Completion (TTC) and Anastomosis Lapse Index (ALI), respectively.

**Key Results:** Fourteen participants (6 novices, 5 plastic surgery specialist trainees and 3 consultants) completed both microsurgical skills sessions. In total, 28-microvascular anastomosis were analyzed. Mean baseline TTC for the group was 20.36 min. With cognitive distraction and external stress mean TTC decreased to 17.87 min. Mean baseline ALI score for the group was 3.32 errors per anastomosis. The introduction of cognitive distraction and external stress increased the mean to 4.86 errors per anastomosis. Total errors per anastomosis increased from 91 errors at baseline to 137 errors with cognitive distraction and external stress. Under stress, participants were more efficient but had reduced anastomotic accuracy.

**Conclusion:** Under stress, surgeons were more efficient, this translated into faster completion of a microsurgical anastomosis. Efficiency, however, came at the expense of accuracy.

## Introduction

Since it was first described in the literature over a century ago microsurgical techniques have become an essential part of plastic surgery practice offering significant advances in soft tissue reconstruction. Microsurgery is now an essential and routine part of plastic surgical practice. The ability to perform microsurgery has thus become a necessity for both trainees and consultants alike. Microsurgery offers a number of uniquely challenging technical elements that can be attributed to the scale on which surgery is performed. Operating on vessels with a caliber of 1–2 mm in diameter under a stereoscopic vision offers little or no haptic feedback, limited dimensional perspective and a need for fine dexterity. These technical elements in combination with the often noisy and distracting theater environment are what makes microsurgery uniquely challenging.

As one would expect there is a steep and unforgiving learning curve associated with microsurgery (1, 2). Critical to success for trainees in microsurgery is advancing through this steep learning curve. A simulation based training approach has been recognized as the most effective and appropriate learning environment in order to attain the learning curve and test technical proficiency in microsurgery (1–11).

In recent years within the wider field of surgery there has been a shift in focus assessing the role and impact of non-technical skills on surgical performance for both trainees and experienced surgeons alike. This is reflected in the growing body of literature advocating movement toward the incorporation and training of cognitive and mental skills curricula focusing on the non-technical skills that aid and often enhance technical surgical skill and performance (9, 12–22). The role of stress, as well as cognitive distraction, and their impact in impairing technical performance is well documented (9, 12–22). Operating room distractions and interruptions (ORDIs) are widely acknowledged as part of the theater environment and relate specifically to occasions where the attention of one or more of the surgical team is drawn away from the primary task toward a less important or case irrelevant task (14). Such distractions have been shown to be more detrimental to the performance of surgeons in the learning curve with the degree of distraction being proportional to the degree of difficulty of the task and technical ability of the surgeon (19, 23). The unique technical and cognitive demands of microsurgery may render trainees, as well as experts, more susceptible to the affects of cognitive distraction and stress. No studies to date have looked at the impact of ORDIs, cognitive distractions and external stressors on the ability to complete cognitive and technical skills in parallel in a microsurgery setting.

The aim of this study was to test the hypothesis that cognitive distraction and external stressors negatively affect microsurgical performance in a high fidelity simulation setting.

## Methods

Ethical approval was obtained from an institutional ethics review board (Galway Clinical Research Ethics Committee, Merlin Park Hospital, Galway, Ireland). Participants written informed consent was obtained prior to inclusion in the study.

Fourteen surgeons were recruited across various levels of training, from complete novice to consultant, all with varying levels of microsurgical experience. The study was conducted during two microsurgical skills sessions, each 1 month apart. Session 1 established baseline microsurgical skill and Session 2, the intervention session, involved the introduction of cognitive distraction and external stress into the microsurgery simulation.

**Figure 1:**
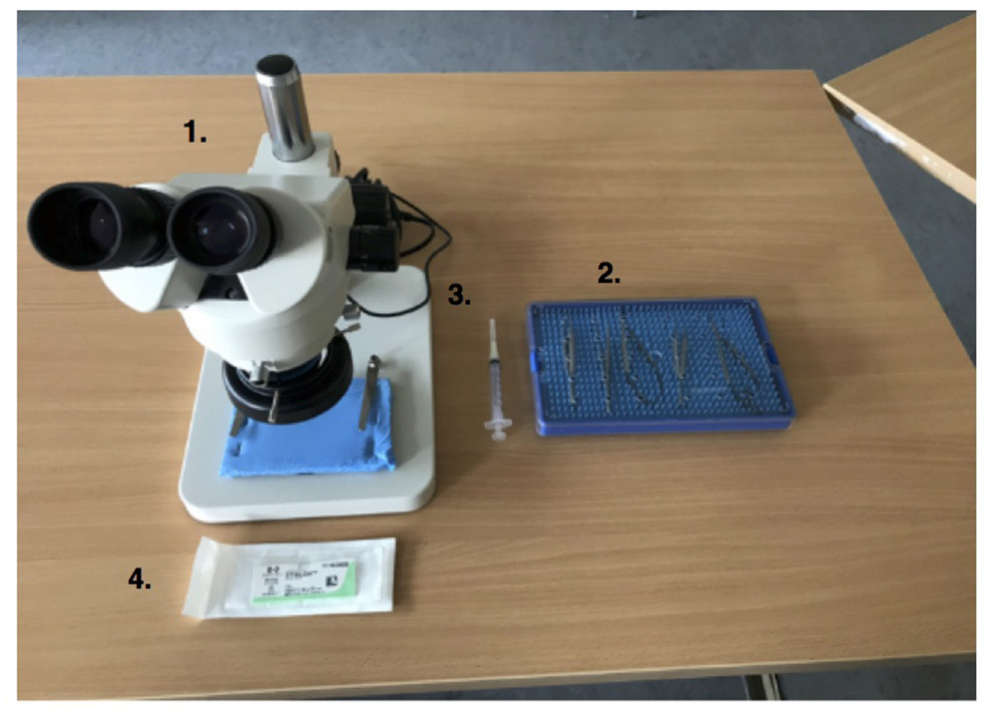
Standardized Microsurgical Workstation; 1. Desktop Microscope 45X Zoom, 2. Microsurgical instruments (micro-needle holder, micro-scissors, microsurgical forceps, vessel dilator), 3. 3 ml syringe with water, 4. 8.0 ethilon™ suture.

A standardized microsurgical workstation and layout was setup identical for each participant (Figure 1). Each participant was given a desktop microscope (45X zoom, 10 cm working distance, www.worldmicrotraining.com), microsurgical instruments (micro-needle holder, micro-scissors, microsurgical forceps, vessel dilator), 3 ml syringe filled with water for injection and an 8.0 ethilon™ suture to complete their task. A pre-dissected non-living, chicken thigh femoral vessel model was used for the purpose of our study (24). The vessel was mounted on a display pad, free of tension and an arteriotomy performed in the center of the vessel.

Each participant was given the same written instructions to execute a well-defined task which entailed placement of 8 interrupted microvascular sutures, evenly placed around the chicken femoral vessel circumference, 4 sutures on the posterior wall and 4 sutures on the anterior wall of the vessel, to result in an accurate microsurgical anastomosis. Participants were allowed to adjust focus and setup to their individual needs prior to the start of the task. Timing started when the first instrument was picked up and stopped when the last suture was placed and all instruments were laid down.

**Figure 2:**
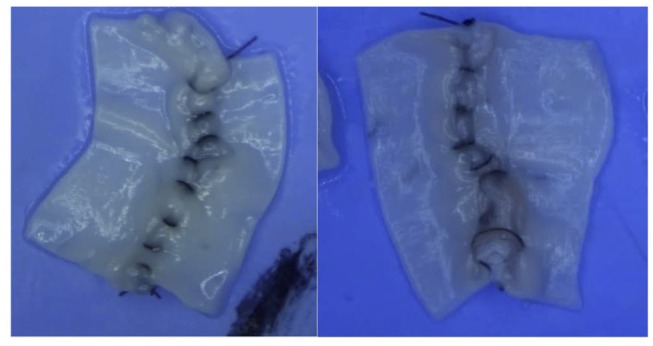
Participant micro-anastomosis; post arteriotomy with intimal wall laid out and visible suture placement.

Upon completion, each anastomosis was cut longitudinally, laid open, and the intimal side photographed at 25X magnification (Figure 2).

**Figure 3:**
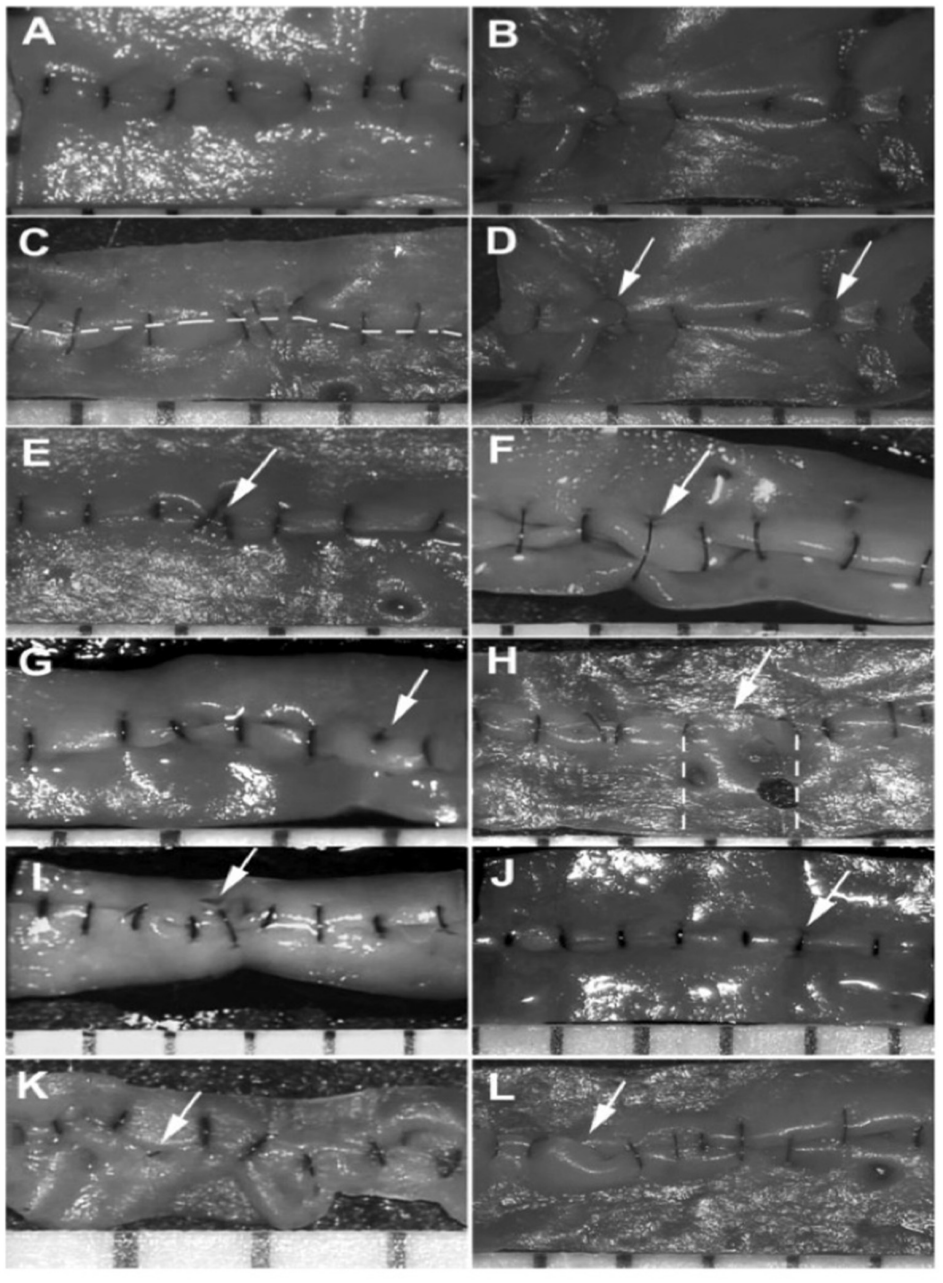
ALI Index: **(A)** Image demonstrating an error free anastomosis. **(B)** An anastomosis with multiple errors, **(C)** Error 1: Disruption of the anastomosis line created by the opposed vessel ends. **(D)** Error 2: Inadvertently catching the back-or- sidewall when taking suture bites causing occlusion of the lumen. **(E)** Error 3: Placing of an oblique stitch causing tissue distortion. **(F)** Error 4: Taking too wide a bite that causes tissue infoldment. **(G)** Error 5: Placing of a stitch that does not go through the full thickness of the vessel. **(H)** Error 6: Unequal distancing of sutures that is more than twice what is expected. **(I)** Error 7: Causing a visible tear in the vessel wall. **(J)** Error 8: Excessively tight suture that strangulates the tissue. **(K)** Error 9: Threads left in the lumen. **(L)** Error 10: Allowing for large edge overlap. Ghanem et al. Anastomosis Lapse Index (ALI): A Validated End Product Assessment Tool for Simulation Microsurgery Training (10).

Two expert external reviewers, blinded to each participant's identity and sequence of anastomoses, reviewed and scored anastomosis photographs for accuracy using the Anastomosis Lapse Index (ALI) (Figure 3) (10).

One month later, in the second session, participants were asked to complete the exact same microsurgical task, however this time they were subject to cognitive interruption and distraction as well as external stressors as they carried out the microsurgical anastomosis. Participants were asked 3 sets of questions at 2 min intervals. Questions were based around operative room distractions and interruptions (ORDIs) (14). ORDIs were based on 3 distracting sets of questions intended to interrupt and distract participants from their task. These questions were: 1. Medically themed arithmetic questions (e.g., calculations of drug dosages) 2. Unrelated controversial political/current affairs themed questions where participants were asked their opinions 3. Personal questions (e.g., why a participant chose their chosen career). The same objective outcome measures were used to assess participants as those in session one, i.e., TTC for efficiency and ALI score for accuracy of microsurgical anastomosis.

Data from both sessions were collected, de-identified, entered into an excel spreadsheet, and stored in secure format. Statistical analysis was carried out using IBM SPSS 24.0 program (2016, NewYork United States of America).

## Results

In total 14 participants were recruited into the study (6 novices, 5 specialist trainees and 3 consultants or expert level microsurgeons). Eight participants had previously undertaken a microsurgical training course. Participant demographics and previous microsurgical experience is illustrated in Table 1. In total 28 microvascular anastomoses were analyzed.

**Table 1:** Participant information on microsurgical experience.

**Sex**	**Stage of training**	**Previous micro course(s)**	**Micro cases at work (per month)**	**Practice of Micro skills (per month)**	**Arterial anastomosis performed (simulated)**	**Arterial anastomosis performed (*in-vivo*)**
M	Novice	0	≤ 1	≤ 1	3	0
M	Novice	0	0	0	0	0
M	Novice	0	0	0	0	0
F	Novice	0	0	0	0	0
M	Novice	0	0	0	1	0
M	Novice	0	0	≤ 1	10	0
F	ST	1	0	≤ 1	5	2
F	ST	1	≤ 1	≤ 1	15	10
F	ST	1	0	≥2	20	3
M	ST	2	0	0	10	10
M	ST	1	≥2	≥2	1	4
M	Consultant	≥2	≥2	0	5	>200
M	Consultant	≥2	≥2	0	20	60
F	Consultant	≥2	≥2	0	20	70

*Novice, ST, Specialist Trainee, Consultant; SIM, Simulation; in-vivo, live patient*.

**Figure 4:**
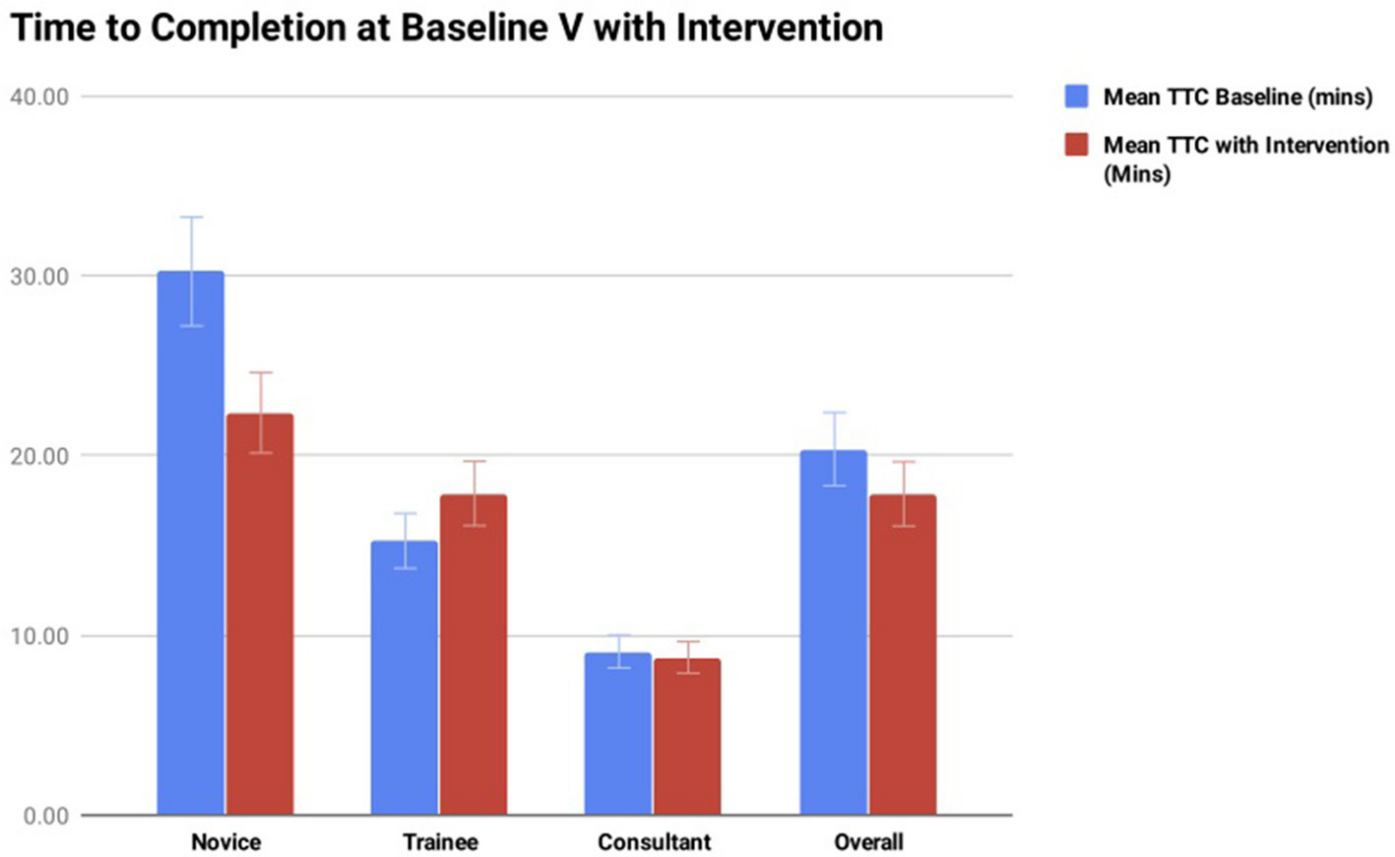
Summary of the TTC of microsurgical anastomosis at baseline vs. TTC with intervention (cognitive distraction and external stress). The breakdown comparison of each subgroup is also illustrated.

At baseline, the group overall had a mean TTC of a microsurgical anastomosis of 20.36 min (range 12.36–39.45 min). With the introduction of cognitive distraction and external stress the mean TTC of a microsurgical anastomosis decreased to 17.87 min (range 10.26–23.09 min). The comparison of TTC without and with cognitive distraction is illustrated in Figure 4. On analysis of each subgroup, at baseline the novice group had a mean TTC of a microsurgical anastomosis of 30.24 min (range 26.43–39.45 min), and with the introduction of cognitive distraction/stress this decreased to a mean TTC of 22.39 min (range 21.25–26.5 min). At baseline, the specialist trainee group had a mean TTC of 15.26 min (range 12.36–17.4 min), and with the introduction of cognitive distraction/stress this increased to a mean TTC of 17.90 min (range 10.26–23.09 min). At baseline, the consultant group had a mean TTC of 13.38 min (range 12.35–14.4 min), and with the introduction of cognitive distraction/stress this decreased to a mean TTC of 13.15 min (range 12.35–13.46 min).

**Figure 5:**
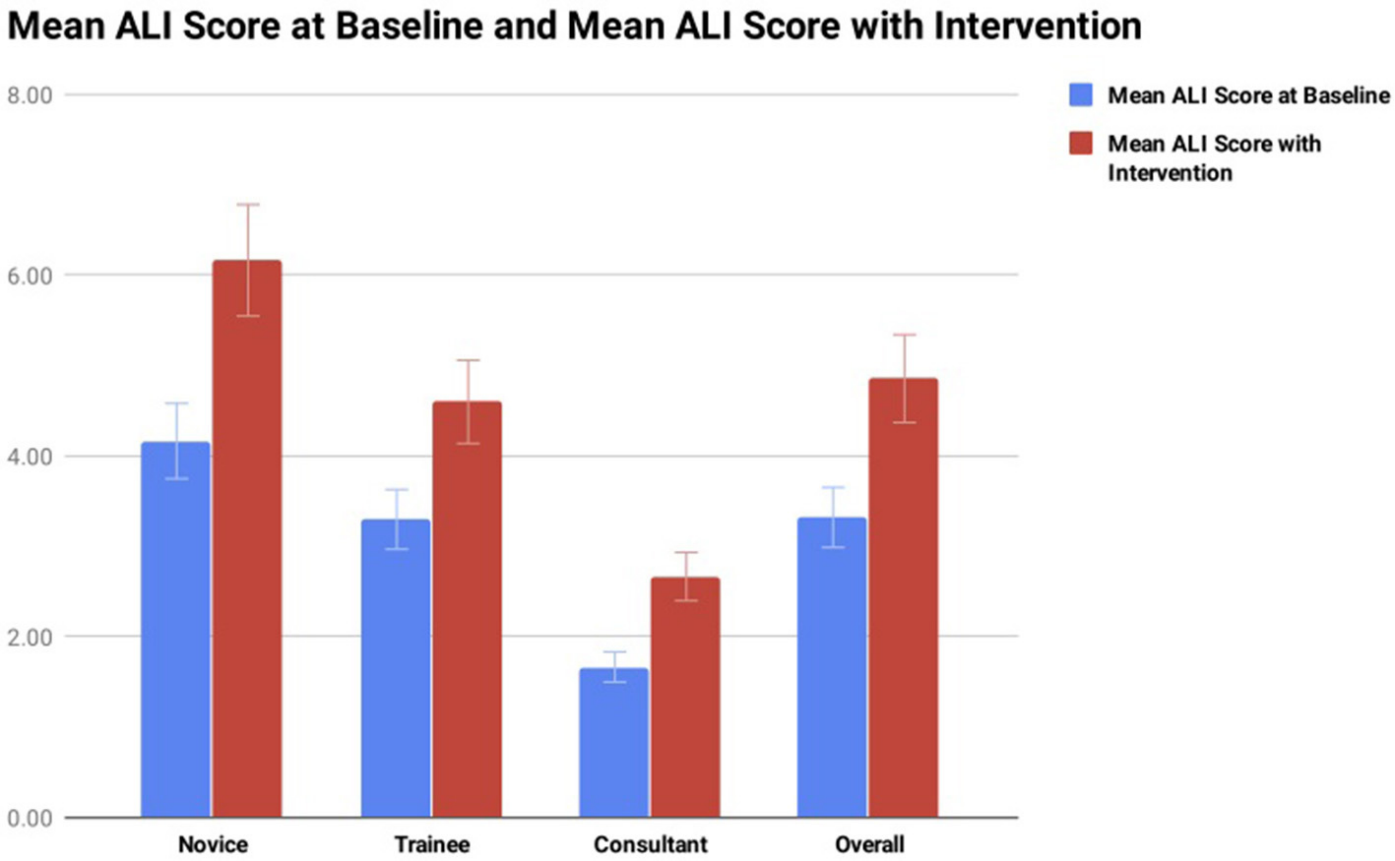
Illustrates the mean ALI score for each group at baseline compared to ALI score with the introduction of cognitive distraction/stress. Lower ALI score equates to more accurate microsurgical anastomosis.

Anastomosis lapse index score was used to assess the number of anastomotic errors identified at baseline compared to the number of anastomotic errors identified following the introduction of cognitive distraction and external stress (Figure 3). At baseline the group overall had a mean ALI score of 3.32 errors per anastomosis (range 1–4.5 errors) (Figure 5). With the introduction of cognitive distraction and external stress the mean ALI score for the group overall increased to 4.86 errors per anastomosis (range 2–7 errors). At baseline, the novice, specialist trainee and consultant groups had mean ALI scores of 4.17 (range 3.5–4.5 errors), 3.30 (range 2.5–4 errors), and 1.67 errors (range 1–2.5) per anastomosis, respectively. All subgroups had an increased ALI score with the introduction of cognitive distraction and external stress, with the novice group increasing their mean ALI score to 6.17 errors (range 5.5–7 errors), the specialist trainee group increasing their ALI score to 4.6 errors (range 4–5.5 errors), and the consultant group increasing their mean ALI score to 2.67 errors (range 2–3.5 errors).

**Figure 6:**
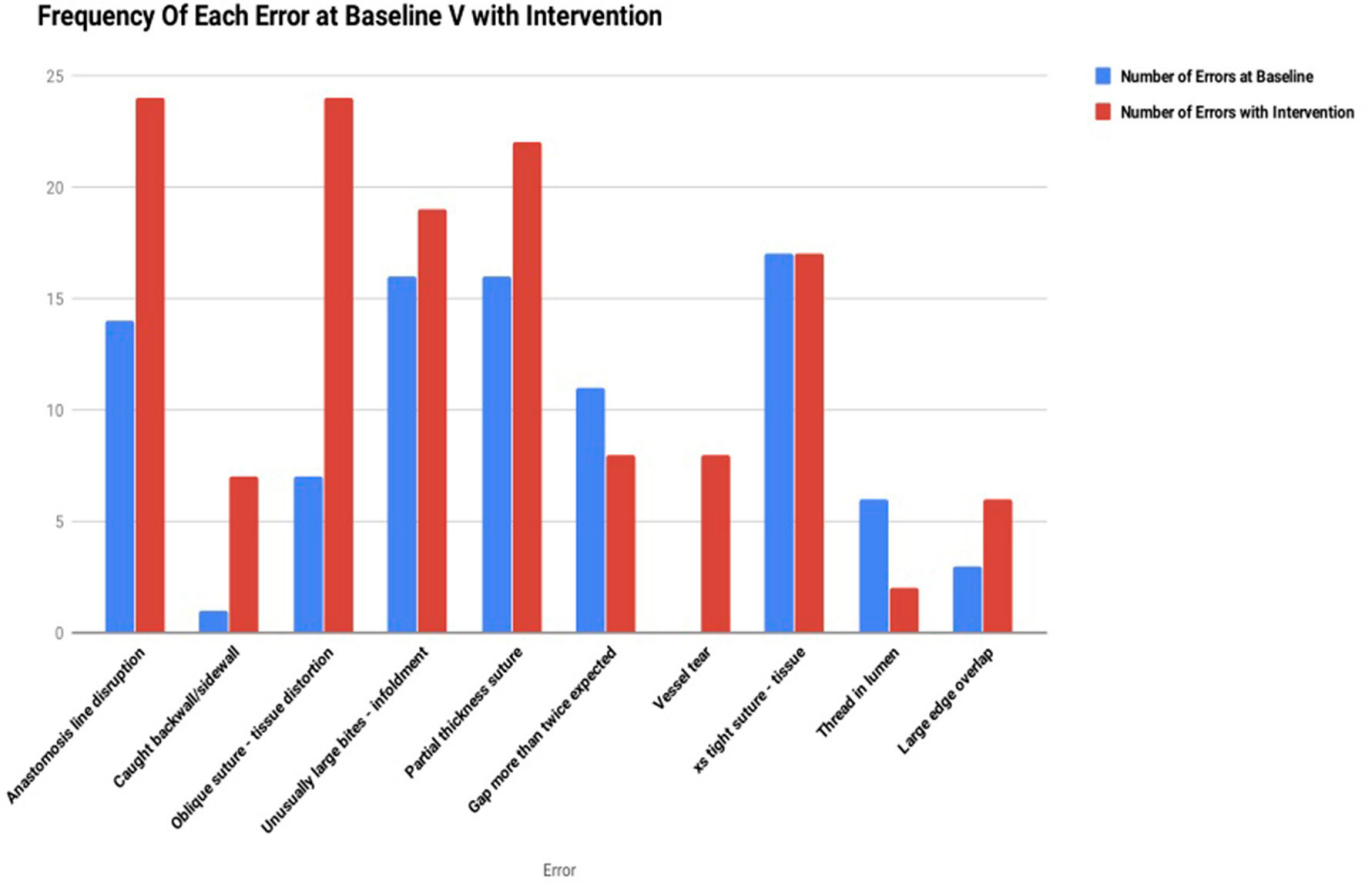
Frequency of each error at baseline vs. with intervention.

**Figure 7:**
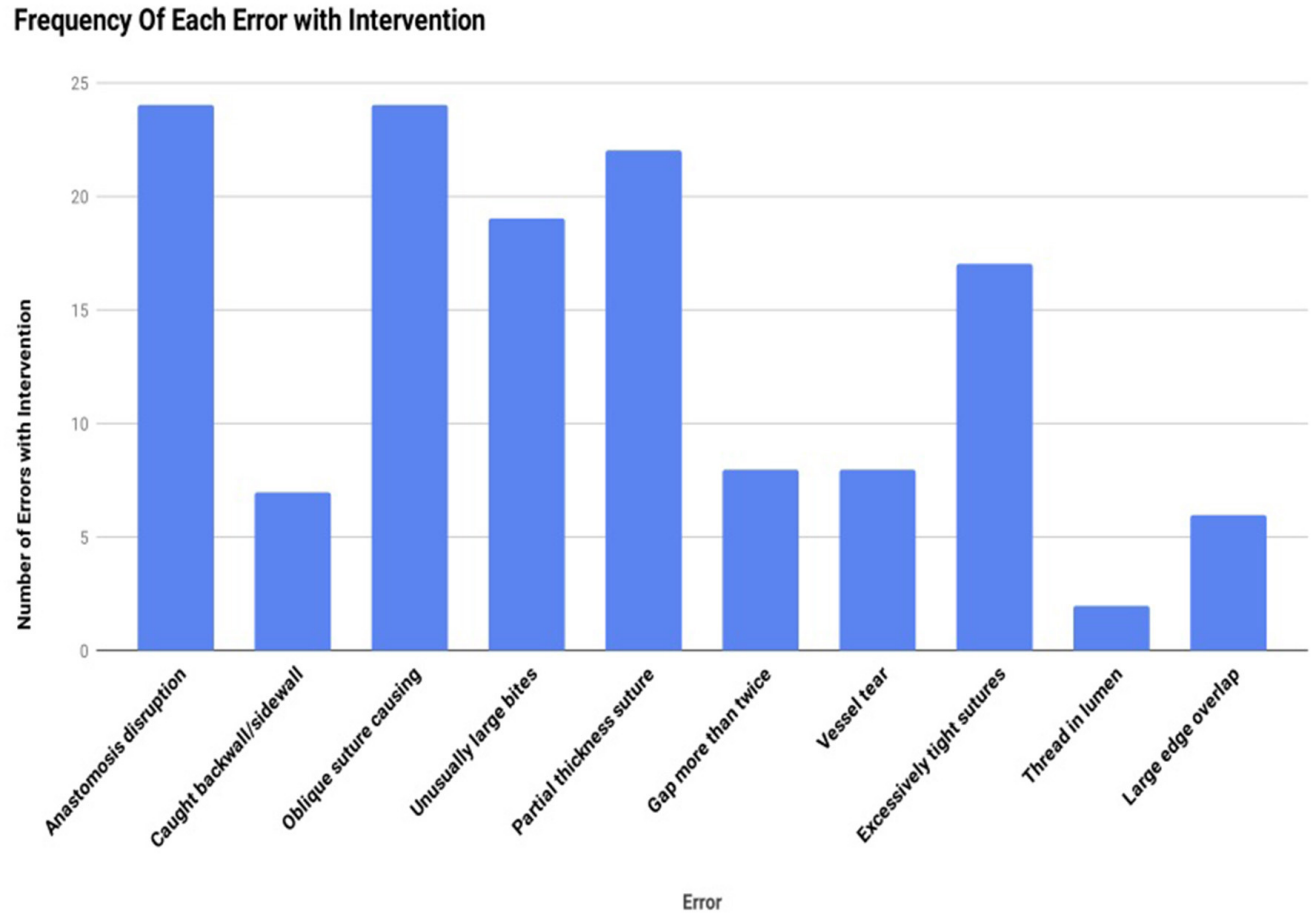
Overall frequency of each anastomotic error with intervention.

Analysis of anastomotic errors (Figure 6) showed that the total number of errors cumulatively increased amongst all subjects from 91 errors per anastomosis at baseline to 137 errors per anastomosis with the introduction of cognitive distraction and external stress. The comparison of pre and post intervention frequency of errors is illustrated in Figure 6. The overall frequency of each error with intervention is illustrated in Figure 7.

An interclass correlation coefficient (ICC) test was carried out to assess inter-observer reliability of our 2 independent blinded reviewers. A two-way mixed effects model evaluating consistency between rater's scores was calculated for each session. The first session had an ICC = 0.71 (*p* = 0.001) and the second session had an ICC = 0.88 (*p* < 0.001) confirming high inter-observer reliability.

## Discussion

The presence and prevalence of distraction and stress in the theater environment is something that cannot be ignored. The detrimental effect of both distraction and stress on surgical performance in general has been widely described (9, 13–19, 22, 25). We believe our findings provide support that mental skills training and taught coping mechanisms for intraoperative stress and distraction should be implemented in a microsurgical training curriculum, as a supplement to technical microsurgical skills training. When developing a surgical simulation model the concept of physical fidelity vs. psychological fidelity is undoubtedly an important one. It has been argued that psychological fidelity is a more important aspect of simulation (8). Stress and cognitive interruption are two very prominent elements that make up the psychological fidelity of a real time operating theater. Both are almost unavoidable in the operating theater environment with distractions occurring as frequently as every 3 min with on average 13.5 interruptions per case (17). Since these elements of the theater environment are unavoidable new microsurgeons need to learn how to focus attention on the microsurgical task at hand while engaging with distractions and stress. Introduction of simulated training with realistic psychological fidelity should enable trainees to develop the appropriate coping strategies.

Stress in the theater setting is believed to greatly influence surgical performance and task execution (9). Increased stress has been shown to negatively effect cognitive functioning, information processing and skills based surgical motion leading to detrimental affects on surgical performance and patient safety (9, 18). High levels of stress have been shown to impair judgment, decision-making, and communication among trainee surgeons (18). Stress in theater has been described as a dynamic phenomenon where a stress cascade occurs with adverse or stress inducing factors leading to progressive stress levels (20). Increased intra operative workload results in increased cognitive demand, increasing to a level that eventually surpasses the trainees/surgeons stress coping ability leading to excessive stress which impacts on the trainees/surgeons ability to perform the surgical task at hand in a safe manner (26). The challenging cognitive demands specific to microsurgery, could and possibly do result in a lower threshold for the triggering of this cascade. Although the current study does not define this threshold, it has succeeded in objectively illustrating that the negative impact of stress on trainee surgeons can be replicated in a microsurgical simulation setting where it had a doubly detrimental effect on both outcome measures, TTC and ALI, in our trainee cohort of participants.

As alluded to already, distraction is also a prominent and unavoidable element of the theater environment. The role of distraction in surgery has been the subject of numerous studies to date however none have examined its affect on microsurgical performance (12, 14, 17, 22, 25). In the literature various distractions encountered in theater have been described such as, case irrelevant communication, equipment issues, phone calls/bleeps, movement and procedural issues (17). Distraction has been shown to be detrimental to numerous objective measurements of surgical proficiency, accuracy and TTC, ultimately translating into poorer patient outcomes in the real operating theater (14–18, 25). The effects of distraction can be dependent on task difficulty (17). It has been shown that there is a task interaction effect with distraction with more complex tasks in the context of simulation in laparoscopic surgery (19–21, 27). As microsurgery is widely recognized as a more complex and technically demanding surgery, one would have to assume that this effect translates to microsurgery. Working on the assumption that the majority of published data relating to the impact of distraction in non-microsurgery translates to the field of simulation in microsurgery, it must be borne in mind that microsurgery is a different surgical entity both technically and in terms of its cognitive demands. The current study, for the first time, demonstrates that many of the aforementioned effects of distraction translate to microsurgery and have an objectively measurable negative impact on microsurgical accuracy.

The ultimate goal of any microsurgical trainee is to attain a level of proficiency whereby they progress from being a novice trainee to an expert. Consultant or expert surgeons interact with stress and cognitive distraction differently when compared to trainees. More experienced surgeons have been shown to be less affected by distractions when performing a surgical skill (17). The concept of automaticity has been attributed as the explanation for this ability to deal with adverse external distraction and stress with little or no affect on performance. Automaticity is where experience in performance of a particular technical skill is thought to lead to a state of automatic performance, requiring less conscious effort or cognitive capacity (17). This in turn allows for increased cognitive capacity to deal with other tasks, not necessarily related to the primary task at hand. Gaining automaticity thus reduces the effect of a distracting or a stressful event (17). The ability to cope with cognitive distraction and stress to the point that it does not hinder or affect a trainee's surgical proficiency reflecting gained automaticity could potentially be used as an objective measure of progress as well as experience. The results herein reiterate the concept of gained automaticity specifically in microsurgery and illustrate that the effect of cognitive distraction and stress among the consultant cohort lead to increased efficiency with a negligible impact on accuracy. The consultant cohort TTC improved by almost 30 s with the introduction of stress and cognitive distraction while their mean ALI only increased by 1 point. These results were a truly differentiating outcome measure between the consultant cohort and the other study participants (trainees and novices).

Interestingly our results showed that there was a reduction in the mean TTC of the group studied from baseline to intervention, 20.36 min at baseline to 17.82 min with the introduction of ORDIs. When this is broken down this can be mostly attributed to a marked improvement in the novice group. We have attributed this improvement to task familiarity and consolidation of technical skills. This group had never performed a microsurgical anastomosis prior to the first session. We observed a marked improvement in technical efficiency from session one to session two even with the presence of ORDIs. Looking at the consultant group there was a more modest improvement in TTC between session one and session two, 13.38 min at baseline to 13.15 min with ORDIs. This we feel highlights the gained automaticity of the consultant or expert group in that the introduction of ORDIs had little to no effect on surgical efficiency. The most interesting group to analyze in relation to TTC is the trainee group as the authors believe this group is reflective of the true effect of cognitive distraction and external stress on microsurgical skill acquisition and the impact of training, as this represents the cohort that is in the microsurgical learning curve where distraction has been shown to have the greatest effect (19).

## Study Limitations

The principle limitation of this study was that it was limited to a very specific type of cognitive distraction and stress that would only form one part of a larger number of possible theater distractions. With higher fidelity simulation models and simulation environments it would be possible to incorporate a larger and more varied number of distractions. Another limitation of this study is the sample size. A larger, multicenter cohort of participants may be needed to gain more statistically significant outcomes in the analysis of cognitive distraction and stress and their affect in microsurgery performance. The authors feel it is also relevant to highlight the marked improvement in TTC from baseline to intervention in the novice group from session one to session two. They have attributed this to task familiarity and consolidation of skills during the interval period between the first and second session. For the majority of novice participants, but not all, the first simulation session was their very first microsurgical training experience.

## Conclusion

The results herein show that although the introduction of cognitive distraction and external stress improved task efficiency, this came at the cost of accuracy and surgical proficiency in a microvascular simulation setting. The study illustrates, for the first time, that cognitive interruption and external stress have an objectively measurable affect on microsurgical performance. A simulation-based approach to teaching cognitive self-management when faced with ORDI's in microsurgery is needed. Simulation based microsurgery courses should incorporate ORDI basic training into their curriculum in order to mediate and mitigate the effects of stress and cognitive distraction amongst trainees.

## Data Availability Statement

The datasets generated for this study are available on request to the corresponding author.

## Author Contributions

SC: primary author. BM: faculty member, assisted in simulation design and set up. NM and AH: departmental consultant, assisted in rating candidates and participated in study. DB: director of simulation laboratory, assisted in simulation design and set up. SP: clinical lead/supervisor, designed study, assisted with running of study and write-up.

### Conflict of Interest

The authors declare that the research was conducted in the absence of any commercial or financial relationships that could be construed as a potential conflict of interest.
